# Effect of a Chlorhexidine Gluconate-Based Oral Rinse Containing Natural Bioflavonoids on Soft Tissue Inflammation and Subgingival *Porphyromonas Gingivalis* Carriage in Patients With Peri-Implant Mucositis

**DOI:** 10.3290/j.ohpd.c_2388

**Published:** 2025-12-02

**Authors:** Nujud Al-Amry, Munerah S. BinShabaib, Shatha S. AlHarthi, Shikhah Binnjefan, Shatha Alnafissah, Kawther Aabed, Nadine Moubayed, Manal Shalabi

**Affiliations:** a Nujud Al-Amry Assistant Professor, Department of Preventive Dental Sciences, College of Dentistry, Princess Nourah Bint Abdulrahman University, Riyadh, Saudi Arabia. Study research and design, conducting the study, and writing the manuscript.; b Munerah S. BinShabaib Professor, Department of Preventive Dental Sciences, College of Dentistry, Princess Nourah Bint Abdulrahman University, Riyadh, Saudi Arabia. Study research and design, conducting the study, and writing the manuscript.; c Shatha S. AlHarthi Professor, Department of Preventive Dental Sciences, College of Dentistry, Princess Nourah Bint Abdulrahman University, Riyadh, Saudi Arabia. Study research and design, conducting the study, and writing the manuscript.; d Shikhah Binnjefan AEGD Resident, King Abdulaziz Medical City, Central Region (KAMC-CR), Ministry of National Guard – Health Affairs, Riyadh, Saudi Arabia. Study research and design, conducting the study, and writing the manuscript.; e Shatha Alnafissah General Practitioner, Riyadh, Saudi Arabia. Study research and design, conducting the study, and writing the manuscript.; f Kawther Aabed Researcher, Department of Biology, College of Science, Princess Nourah bint Abdulrahman University, Riyadh, Saudi Arabia. Study research and design, conducting the study, and writing the manuscript.; g Nadine Moubayed Researcher, Department of Botany and Microbiology, King Saud University, Science College (female campus), Riyadh, Saudi Arabia. Study research and design, conducting the study, and writing the manuscript.; h Manal Shalabi Consultant, Department of Preventive Dental Sciences, College of Dentistry, Princess Nourah Bint Abdulrahman University, Riyadh, Saudi Arabia. Study research and design, conducting the study, and writing the manuscript.

**Keywords:** bioflavonoids, chlorhexidine, mechanical debridement, mouthwash, peri-implant mucositis, Porphyromonas gingivalis

## Abstract

**Purpose:**

This parallel-group clinical study evaluated whether adding natural bioflavonoids to 0.12% chlorhexidine (CHX) mouthwash enhances outcomes of non-surgical therapy for peri-implant mucositis. The primary aim was to assess soft-tissue inflammatory changes and subgingival *Porphyromonas gingivalis* (*P. gingivalis*) carriage compared with CHX alone.

**Materials and Methods:**

Thirty-three patients were enrolled (test, n = 16; control, n = 17). All patients underwent mechanical debridement, following which patients in the test group were prescribed a bioflavonoid-enriched CHX, whereas those in the control group were prescribed 0.12% CHX. All participants were instructed to rinse their oral cavity every 12 hours for 30 s with their respective oral rinse for 3 weeks. A follow-up clinical evaluation was done after 6 weeks. Peri-implant indices, modified plaque index (mPI), modified bleeding index (mBI), and probing depth (PD) were recorded at baseline and follow-up. Subgingival oral biofilm samples were also collected and assessed for the presence of *P.gingivalis* in both groups. Statistical significance was set at P <0.05.

**Results:**

At baseline, groups were comparable across clinical parameters. At 6 weeks, the test group exhibited significantly lower mPI, mBI, and PD versus controls (all P <0.05). Specifically, mean PD was 2.2 ± 0.04 mm in the test group versus 2.5 ± 0.03 mm in controls; mPI and mBI also favoured the test rinse (0.38 ± 0.04 vs 0.30 ± 0.07; 0.28 ± 0.05 vs 0.32 ± 0.02, respectively). *P. gingivalis* was detected at baseline in all participants; at 6 weeks, carriage persisted in 3/16 (18.8%) test versus 9/17 (52.9%) control patients.

**Conclusion:**

Supplementing 0.12% CHX with bioflavonoids provides added clinical benefits when used as an adjunct to mechanical debridement (MD) in patients with peri-implant mucositis. This combined approach appears more effective than 0.12% CHX alone in reducing peri-implant soft-tissue inflammation and *P. gingivalis* carriage, indicating that bioflavonoid-enriched formulations may represent a valuable therapeutic option in routine clinical practice for improving peri-implant health.

Peri-implant mucositis is a reversible inflammatory condition characterised primarily by bleeding on gentle probing, often accompanied by erythema and oedema, without radiographic evidence of crestal bone loss (CBL).^14^ Contemporary epidemiology indicates that peri-implant mucositis is highly prevalent, affecting roughly half or more of implant patients, with patient-level estimates commonly ranging from approximately 40% to at least 60%, depending on diagnostic thresholds and populations studied.^13^ If not identified and managed in a timely manner, peri-implant mucositis may progress to peri-implantitis, a destructive inflammatory condition characterised by progressive CBL.^31^ This may ultimately lead to implant mobility and, in severe cases, complete implant failure.^29^ Accordingly, early diagnosis and prompt intervention in peri-implant mucositis are critical for mitigating the risk of such complications and preserving the long-term stability and function of dental implants.^26–28,32^

Non-surgical mechanical debridement (MD) remains the primary modality for the management of peri-implant mucositis.^3,11,12,16,34^ However, adjunctive chemotherapeutic agents have been investigated to enhance biofilm control and reduce microbial load, particularly for patients with suboptimal mechanical plaque control.^3,4,16,26,30,33^ Chlorhexidine gluconate (CHX) is considered the gold standard among antimicrobial oral rinses due to its broad-spectrum activity, substantivity, and well-documented efficacy in reducing plaque and gingival inflammation.^4,9,24^ Nevertheless, long-term CHX use has been associated with adverse effects such as tooth staining, taste alteration, and, rarely, mucosal irritation, warranting the exploration of novel formulations that retain antimicrobial efficacy while minimising side effects.^2,6,7^ In a randomised controlled trial (RCT), Al-Zawawi et al^2^ assessed the anti-inflammatory efficacy of a herbal-based mouthwash (HM) in controlling periodontal soft-tissue inflammation. The results showed that HM possess anti-inflammatory properties and can successfully be used for the management of periodontal inflammation.^2^ Similarly, another RCT5 reported that herbal mouthwashes are as effective as 0.12% CHX for the management of peri-implant mucositis. Natural bioflavonoids, polyphenolic compounds widely distributed in fruits, vegetables, and certain medicinal plants, exhibit antimicrobial, antioxidant, and anti-inflammatory properties that could complement the activity of 0.12% chlorhexidine (CHX).^35,36^ Studies^10,22,23^ have shown that some of these inhibit *Porphyromonas gingivalis* (*P. gingivalis*) growth, disrupt biofilm formation, and attenuate lipopolysaccharide-induced inflammatory responses in host cells. While studies^5,9,24^ have assessed 0.12% CHX alone as an adjunct to MD in peri-implant mucositis therapy, there is limited clinical evidence on the efficacy of 0.12% CHX formulations enriched with natural bioflavonoids.

This study aimed to evaluate the clinical and microbiological effects of a 0.12% CHX-based oral rinse containing natural bioflavonoids on soft-tissue inflammation and subgingival *P. gingivalis* carriage in patients with peri-implant mucositis. It is hypothesised that adding bioflavonoids to 0.12% CHX mouthwash enhances its effectiveness by working synergistically to suppress *P. gingivalis* and reduce peri-implant tissue inflammation in patients with peri-implant mucositis.

## MATERIALS AND METHODS

### Ethical Approval

The present study was reviewed and approved by the Research Ethics Committee at the Princess Nourah bint Abdulrahman University, Riyadh, Saudi Arabia (Approval # HAP-01-R-059). Participation was entirely voluntary, and all individuals were requested to read and sign a written informed consent form. The patients were also informed that they could withdraw from the study at any stage without penalty or consequence. All participants were invited to ask questions.

### Inclusion and Exclusion Criteria

The inclusion criteria were as follows: (a) individuals ≥18 years old; (b) patients had at least one functional dental implant *in situ* for a minimum of 1 year; (c) patients diagnosed with peri-implant mucositis; (d) signing the written informed consent form. The following exclusion criteria were entailed: (a) habitual tobacco smoking and use of electronic nicotine delivery systems; (b) use of smokeless tobacco products; (c) habitual alcohol use; (d) individuals with or with a history of periodontitis within the past 60 months; (e) patients diagnosed with peri-implantitis. Moreover, patients with self-reported systemic diseases such as diabetes mellitus (DM), cardiovascular disorders, hepatic and/or renal diseases and patients with HIV/AIDS were excluded. Pregnant and/or nursing females were also excluded.

### Definition of Peri-Implant Mucositis

Peri-implant mucositis was defined as the presence of inflammation in the peri-implant soft tissues, characterised clinically by bleeding and/or suppuration on gentle probing, with a peri-implant PD threshold of ≥4 mm, in the absence of any radiographic evidence of CBL beyond the initial remodelling phase.^4,8^

### Questionnaire

In this study, a structured, self-administered questionnaire was designed to collect demographic information relevant to the participants’ dental implant history. The questionnaire included both closed-ended and short-answer items, with sections on (1) age (recorded in completed years), (2) gender (male/female/other), and (3) duration of implants in function (recorded in years). The instrument was developed following a review of existing validated questionnaires in implant dentistry and was pre-tested on a sample of 10 individuals to ensure clarity, relevance, and comprehensiveness. Participants were provided with written instructions for completing the questionnaire, and assistance was offered if clarification was needed.

### Assessment of Patients’ Digital Dental Records

An experienced investigator reviewed the digital dental records of all eligible patients to extract relevant clinical information. The following information was retrieved from the dental records: (a) total number of implants placed in each patient along with the anatomical location of each implant (maxilla or mandible); (b) implant insertion torque values in Newton centimetres (Ncm); (c) type of implant-retained prosthesis (cement-retained or screw-retained). The speciality of the operating clinician (oral and maxillofacial surgeon, periodontist, prosthodontist, or general dentist) was identified from the provider profile recorded in the electronic dental record system. All extracted data were cross-verified against operative notes, radiographs, and prosthetic design records when available, to ensure accuracy and completeness. Only complete and verifiable entries were included in the final analysis to minimise information bias.

### Collection of Oral Biofilm Samples from the Peri-Implant Subgingival Biofilm

A collection of subgingival oral biofilm (OB) samples was performed preoperatively (at baseline) and after 6 weeks. In summary, peri-implant tissues were isolated with cotton rolls, and the site was gently dried with oil-free air for approximately 5 s. Supragingival plaque deposits were carefully removed using a sterile curette, avoiding subgingival disruption. Sterile paper points (ISO size 40–60) were inserted in parallel to the long axis of the implant to ~1 mm short of the pocket base and left in place for 10 s to absorb subgingival biofilm and crevicular fluid. Two to three paper points were obtained per site and were pooled into a single sample.^15^ Immediately after retrieval, paper points were transferred into sterile microcentrifuge tubes containing 1.0 ml Tris-EDTA buffer (pH 8.0) or equivalent nucleic-acid stabiliser. Samples were kept on ice in the clinic and stored at −80°C for up to 2 h until DNA extraction.^37^ The DNA was extracted using a silica-membrane kit with mechanical lysis. Pathogen-specific quantification of *P. gingivalis* was performed by TaqMan qPCR (16S rRNA gene), with standard curves prepared from reference strains; broader community profiling used 16S rRNA gene sequencing (V3–V4).^38^ All laboratory-based investigations were performed by a trained, calibrated and blinded investigator.

### Management of Peri-Implant Mucositis

Peri-implant mucositis was managed using a standardised non-surgical MD protocol.^30^ A single calibrated clinician performed all procedures to ensure consistency. After isolating the treatment area and providing adequate illumination, supra- and submucosal biofilm removal was carried out using titanium curettes (Hu-Friedy, Chicago, IL, USA) specifically designed for implant maintenance, in combination with a low-abrasive glycine powder air-polishing system (AIRFLOW®, EMS, Nyon, Switzerland) for biofilm disruption along the implant surface and peri-implant sulcus.^29^ The peri-implant sulcus was instrumented circumferentially with plastic curettes (Hu-Friedy, Chicago, IL, USA), applying light pressure to avoid damage to the titanium surface, until tactile and visual assessment indicated a smooth, biofilm-free surface. Following mechanical instrumentation, the peri-implant tissues were gently irrigated with sterile saline to remove residual debris. Oral hygiene instructions were reinforced, including the use of a soft-bristled toothbrush, interdental brushes with plastic-coated wire, and antimicrobial mouthrinse. All patients were scheduled for a 6-week follow-up.

### Study Groups, Randomisation and Allocation Concealment

Following mechanical debridement, participants were randomly allocated to two parallel groups according to the type of mouthwash prescribed for postoperative care. Randomisation was performed using a computer-generated random sequence to ensure equal allocation and minimise selection bias (IBM SPSS Statistics for Windows, Version 29.0, IBM, Armonk, NY, USA). Allocation concealment was maintained by placing the group assignments in sequentially numbered, opaque, sealed envelopes that were opened only after the debridement procedure was completed. In the control group, participants were instructed to rinse twice daily for 3 consecutive weeks with 15 ml of a non-alcoholic 0.12% CHX solution. In the test group, participants were instructed to rinse twice daily with 15 ml of 0.12% CHX containing natural bioflavonoids. All participants were advised to avoid eating, drinking, or rinsing with water for at least 30 min following each use to maximise the substantivity of the mouthwash.

### Assessment of Peri-Implant Clinical Parameters

All peri-implant clinical parameters were recorded by a blinded and calibrated examiner (kappa score 0.85) using a plastic periodontal probe (UNC-15, Hu-Friedy, Chicago, IL, USA). The measurements were taken at four sites per implant (mesial, distal, buccal, and lingual/palatal). The mPI and mBI were assessed according to the criteria described by Mombelli et al.^25^ Briefly, the mPI was scored as follows: Score 0: No detection of plaque; Score 1: Plaque recognised only by running a probe across the smooth marginal surface of the implant; Score 2: Plaque visible to the naked eye; and Score 3: Abundant plaque accumulation. The mBI was recorded within 30 s of probing and scored as follows: 0 = no bleeding; 1 = isolated bleeding spots; 2 = confluent red line of blood along the mucosal margin; and 3 = profuse bleeding. The peri-implant PD was measured at six sites per implant using a graded plastic periodontal probe (UNC-15, Hu-Friedy, Chicago, IL, USA), positioned parallel to the long axis of the implant, from the mucosal margin to the base of the peri-implant sulcus or pocket, to the nearest millimetre (mm).^3,4^ These parameters were re-assessed at 6 weeks of follow-up.

### Sample Size Estimation

The target sample size was estimated based on data derived from a pilot investigation. Power calculations were conducted using G*Power v3.1.9.7 (Heinrich-Heine-Universität Düsseldorf, Germany). The prespecified primary endpoint was the change in peri-implant soft-tissue inflammatory status, measured as probing depth (PD) at the implant level, from baseline to the end of the rinsing period. A key secondary endpoint was the proportion of sites positive for subgingival *P. gingivalis* at follow-up. The statistical model was set to a two-sided t-test for independent means, with an effect size (Cohen’s d) derived from the pilot data corresponding to an anticipated 2 mm reduction in peri-implant PD. With a significance level (α) of 0.05 and a desired power (1-β) of 0.82, the analysis indicated that a minimum of 15 individuals per group (test and control) would be required to detect the hypothesised difference (estimated power 82%). To account for potential dropouts or missing data, recruitment slightly exceeded the calculated minimum.

### Statistical Analysis

Statistical analyses were performed using IBM SPSS Statistics, Version 29.0 (IBM Corp., Armonk, NY, USA) and R, Version 4.3.2 (R Foundation for Statistical Computing, Vienna, Austria). Normality was assessed for each continuous endpoint (eg, soft-tissue indices and quantitative *P. gingivalis* counts) using the Shapiro–Wilk test. The primary comparison between test and control groups at the end of follow-up used an analysis of covariance (ANCOVA) with the follow-up value as the dependent variable, group as the fixed factor, and the corresponding baseline value as a covariate to improve precision and account for any baseline imbalance. As a complementary analysis, between-group differences were also evaluated on (i) raw follow-up values and (ii) change-from-baseline (Δ = follow-up–baseline): if normally distributed with equal variances, by independent-samples t-tests; otherwise by Mann–Whitney U tests. Within-group changes from baseline were assessed with paired t-tests (normal data) or Wilcoxon signed-rank tests (non-normal/ordinal data). All analyses were two-sided with α = 0.05.

## RESULTS

### General Characteristics

In total, 33 patients with peri-implant mucositis were included. Seventeen and 16 individuals were included in the control and test groups, respectively. There was no significant difference in the mean age of all individuals. All implants (n = 33) had replaced missing first or second molars. Twenty-two and 11 implants were in the posterior maxilla and mandible, respectively. The implants were in function for 4.8 ± 2.1 years (Table 1). All implants (n=33) were placed 2 mm subcrestally by expert clinicians and had been restored with screw-retained restorations (Table 1).

**Table 1:** Characteristics of the study cohort

Parameters	All patients	Test group	Control group
Participants	33	16	17
Gender (Male : Female)	19 : 14	10 : 6	9 : 8
Mean age in years	51.2 ± 7.4 years	53.2 ± 6.7 years	46.5 ± 5.8 years
Duration of implants in function	4.8 ± 2.1 years	5.1 ± 2.2 years	4.6 ± 1.5 years


### Peri-Implant Clinical Status

At baseline, there was no statistically significant difference in peri-implant mPI, mBI and PD among individuals in the test and control groups. At 6 weeks of follow-up, mPI (P <0.05), mBI (P <0.05) and PD (P <0.05) were significantly higher in the control than in the test group (Table 2). There was no statistically significant correlation between age and gender and peri-implant mPI, mBI and PD.

**Table 2:** Peri-implant clinical status at baseline and 6 weeks of follow-up

Peri-implant parameters	Baseline	6 weeks of follow-up
All patients	Test group	Control group	All patients	Test group	Control group
Total implants in function	33	16	17	33	16	17
Modified plaque index	2.79 ± 0.04^*^	2.82 ± 0.2^†^	2.77 ± 0.05^‡^	0.35 ± 0.05	0.38 ± 0.04	0.3 ± 0.07
Modified bleeding index	2.82 ± 0.08^§^	2.84 ± 0.05^║^	2.8 ± 0.04^¶^	0.3 ± 0.02	0.28 ± 0.05	0.32 ± 0.02
Probing depth	5.1 ± 0.2 mm^#^	4.8 ± 0.07 mm^**^	5.3 ± 0.1 mm^††^	2.3 ± 0.06 mm	2.2 ± 0.04 mm	2.5 ± 0.03 mm
CHX: chlorhexidine gluconate; HMW: Herbal mouthwash; mm: Millimetres ^*^ Compared with all patients at 6 weeks of follow-up (P <0.05) ^†^ Compared with the test group at 6 weeks of follow-up (P <0.05) ^‡^ Compared with the control group at 6 weeks of follow-up (P <0.05) ^§^ Compared with all patients at 6 weeks of follow-up (P <0.05) ^║^ Compared with the test group at 6 weeks of follow-up (P <0.05) ^¶^ Compared with the control group at 6 weeks of follow-up (P <0.05) ^#^ Compared with all patients at 6 weeks of follow-up (P <0.05) ^**^ Compared with the test group at 6 weeks of follow-up (P <0.05) ^††^ Compared with the control group at 6 weeks of follow-up (P <0.05)

### Identification of *Porphyromonas Gingivalis* in the Oral Biofilm at Baseline and Follow-Up

At baseline, *P. gingivalis* was identified in subgingival OB samples collected from all patients with peri-implant mucositis. At 6 weeks of follow-up, *P. gingivalis* was identified in nine (52.9%) and three (18.8%) patients in the control and test groups, respectively. There was no statistically significant correlation between age and gender, and carriage of *P. gingivalis* in the OB.

## DISCUSSION

In the present study, individuals in the test and control groups received standardised conventional treatment (that is, MD) for the management of peri-implant mucositis; however, the 6-month follow-up results showed that adjunctive rinsing with 0.12% CHX enriched with natural bioflavonoids yielded superior clinical and microbiological benefits in terms of reduction in peri-implant soft-tissue inflammation and presence of *P. gingivalis* in the subgingival OB, respectively, than CHX alone. Explicitly, individuals in the test group demonstrated significantly lower scores of mPI, mBI, and PD at follow-up, alongside a markedly reduced carriage of *P. gingivalis* (18.8% vs 52.9% in controls). These data indicate that supplementing CHX with bioflavonoids confers added value beyond the well-established substantivity and broad-spectrum antisepsis of CHX in peri-implant mucositis care. Mechanistically, several complementary pathways could explain the observed effects. First, bioflavonoids possess direct anti-*P. gingivalis* activity and anti-biofilm actions that can augment CHX.^21^ This seems to be a clarification for the significantly lower scores of mPI and mBI in the test than in the control group at follow-up. The authors of the present clinical investigation applaud the results from an *in vitro* study,^21^ which showed that oral rinses consisting of CHX plus bioflavonoids are more efficient against periodontopathogens (such as *P. gingivalis*) than formulations based on CHX alone. One reasoning in this regard is that bioflavonoids inhibit gingipains, cysteine proteases central to *P. gingivalis* virulence, tissue invasion, and nutrient acquisition and suppress OB development, thereby diminishing colonisation pressure and facilitating host resolution of inflammation. Moreover, it has also been reported *in vitro* ^35,36^ that certain plant alkaloids/polyphenols enhance CHX activity against biofilm-associated pathogens, potentially by altering cell envelope permeability, disrupting extracellular polymeric substances, or interfering with efflux, thereby improving CHX access to microbial targets within mature OB. Furthermore, the present clinical reductions in PD are consistent with the host-modulatory properties of polyphenols. Beyond antibacterial effects, flavonoids attenuate LPS-induced cytokine release and oxidative stress (including interleukin 1-beta and tumour necrosis factor-alpha), limiting microvascular changes and connective-tissue matrix degradation that underpin bleeding and pocketing.^23^ When layered on CHX’s plaque-suppressive substantivity, such anti-inflammatory actions likely contributed to the superior soft-tissue response in the test group.^4,23^ From a translational perspective, the dual targeting of (i) biofilm virulence (*P. gingivalis* load and gingipain activity) and (ii) the soft-tissue inflammatory milieu offers a biologically coherent explanation for the magnitude of change observed in both bleeding and PD.^22,23^ While the present study was not designed to measure inflammatory mediators, prior studies^2,6,7^ have shown that plant-derived agents can reduce salivary/pro-inflammatory markers after non-surgical therapy, supporting a host-modulatory contribution to clinical improvement.

The present study has limitations that warrant consideration. The follow-up interval (6 weeks) is relatively short; the durability of the clinical and microbiological gains beyond the early healing phase is unknown. It is well-known that systemic diseases (such as poorly controlled DM) and habitual smoking are risk factors for periodontal and peri-implant diseases.^1,3,16–20^ In this context, it is likely that the efficacy of MD with adjunct CHX (with or without adjuvant bioflavonoids) is compromised in tobacco smokers and patients with immunosuppression. Although laboratory methods included quantitative qPCR in the protocol, the reported microbiological endpoint was dichotomous carriage of *P. gingivalis*; quantitative loads or broader community shifts (eg, *Fusobacterium nucleatum* co-aggregation) were not presented, potentially underestimating microbiological change. In summary, the anti-biofilm potential of bioflavonoid-enriched oral rinses is largely speculative and based on experimental investigations. It is also noteworthy that all implants assessed were placed in single molar sites, which restricts generalisability to the anterior aesthetic zone. Furthermore, all implants were placed by experienced clinicians; therefore, the generalisability of the results to general dental practice is limited. This warrants the need for additional well-designed and power-adjusted clinical trials.

## CONCLUSION

Supplementing 0.12% CHX with bioflavonoids provides added clinical benefits when used as an adjunct to MD in patients with peri-implant mucositis. This combined approach appears more effective than 0.12% CHX alone in reducing peri-implant soft-tissue inflammation and *P. gingivalis* carriage, indicating that bioflavonoid-enriched formulations may represent a valuable therapeutic option in routine clinical practice for improving peri-implant health.

### Acknowledgements

The authors thank Ayman Abdelbaky (Subsidiary of Curaden, Kriens, Switzerland), Executive Director, Curaden Arabia, Riyadh, Saudi Arabia, for their support.

#### Ethics approval and consent to participate

The present study was reviewed and approved by the Research Ethics Committee at the Princess Nourah bint Abdulrahman University, Riyadh, Saudi Arabia (Approval # HAP-01-R-059). Participation was entirely voluntary, and all individuals were requested to read and sign a written informed consent form. The patients were also informed that they could withdraw from the study at any stage without penalty or consequence. All participants were invited to ask questions.

#### Funding

The authors would like to thank the Princess Nourah bint Abdulrahman University Researchers Supporting Project Number (PNURSP2025R63), Princess Nourah bint Abdulrahman University, Riyadh, Saudi Arabia.

#### Conflict of interest statement

The authors declare that they have no conflict of interest.
